# Initiation of prolyl cis-trans isomerisation in the CDR-H3 loop of an antibody in response to antigen binding

**DOI:** 10.1038/s41598-017-16766-8

**Published:** 2017-12-05

**Authors:** Keiko Shinoda, Hideaki Fujitani

**Affiliations:** Laboratory of Systems Biology and Medicine, Research Center for Advanced Science and Technology, The University of Tokyo, 4-6-1 Komaba, Meguro-ku, Tokyo, 153–8904 Japan

## Abstract

Proline cis-trans isomerisation is a regulatory mechanism used in a range of biological processes, and is related to various diseases such as Alzheimers disease and cancer. However, the details of the exact molecular mechanism by which it occurs are not known. Using X-ray crystallography, proline isomerisation has been shown to occur following formation of an antigen-antibody complex between the target epiregulin (EPR) and the antibody 9E5, at proline (Pro103), located in the third complementarity-determining region (CDR) of the heavy chain of 9E5. To obtain an accurate description of the pathway involved in cis-trans isomerisation in this system, we performed ten independent long molecular dynamics (MD) simulations starting at a stable transient bound structure obtained from many short binding MD simulations. As a result, we were able to describe the process by which cis-trans isomerisation is initiated, and suggest a catalysis mechanism for cis-trans isomerization in this antigen-antibody system. We found that Asp102, which is immediately adjacent to Pro103, rotates while changing its interacting partner residues in the light chain of 9E5, and at the same time EPR polar residues help to stabilise the intermediate states in the isomerisation process by interacting strongly with Asp102.

## Introduction

Among the 20 amino acids, proline is unique because both the cis and trans conformers of the prolyl peptide bond are thermodynamically feasible, in contrast to non-prolyl peptide bonds that strongly favour remaining in the trans conformation. Because proline can convert between the cis and trans conformers, it has been shown to function as a molecular timer or switch^1–5^. This prolyl cis-trans isomerisation or “proline switch” is known to be an effective regulatory mechanism in a wide range of biological processes including cell signalling^5–15^, ion channel gating^16,17^, neurodegeneration^18^, gene expression^19,20^, and others^21–24^. Anomalies in these control mechanisms have been suggested to be related to various diseases such as Alzheimer’s disease and cancer^7,11,18,25–29^. Because prolyl cis-trans isomerisation involves rotation around the prolyl peptide bond (i.e. the C-N bond), which is a partial double bond, this requires a high activation energy (approximately 20 kcal/mol)^30^. As a result, cis-trans isomerisation is a slow process occurring over seconds to minutes^31–33^. Cis-trans peptidyl prolyl isomerases (PPIases) are a class of enzymes that catalyse prolyl cis-trans isomerisation to reduce the isomerisation time roughtly by 10^5^ times^34,35^. Three families of PPIases have been identified: Cyclosporin A binding cylophilins, such as cyclophilin A (CypA), FK506 binding proteins (FKBPs), such as FKBP12, and parvulin-like PPIases, such as Pin1. Many studies have been performed to elucidate the catalytic mechanism of PPIases^3^, including not only experimental studies, but also computational studies with free energy calculations using either standard molecular dynamic (MD) simulations^36–40^, QM/MM^41–44^ or accelerating MD simulations^10,45–52^ such as metadynamics, which use a time-dependent biasing potential acting on certain prechosen chemical reaction coordinates. In general, there is a consensus that acceleration of the cis-trans isomerisation rate by PPIases occurs by a catalytic mechanism^53^ in which the substrate is stabilized in its transition state^53,54^. However, the details of the mechanism at the atomic level still remain to be clarified.

Recently, using X-ray crystallography, cis-trans isomerisation was found to occur upon binding of an antibody (9E5) to epiregulin (EPR)^55^. Epiregulin is a member of the epidermal growth factor (EGF) family that, upon binding to the EGF receptor (EGFR), stimulates proliferative signalling in cancer cells. 9E5 is an anti-EPR antibody that inhibits EGFR signalling by EPR but not by EGF^56^. The antigen-binding site of the antibody is formed by six loops referred to as complementary determining regions (CDR), with three (L1, L2, and L3) from the light chain variable domain (VL), and three (H1, H2, and H3) from the heavy chain variable domain (VH). Among the loops, the CDR-H3 loop plays a distinctive role in antigen recognition. Kado *et al*. found that prolyl cis-trans isomerisation occurred in the CDR-H3 loop (Arg98-Gly99-Gly100-Gly101-Asp102-Pro103-Val104-Phe105-Val106-Tyr107-Trp108) of the fragment antigen-binding portion (Fab) of 9E5 in the X-ray crystal structure^55^. The 9E5 Fab has a proline at residue 103 (Pro103) in the CDR-H3 loop, which was found to be in the cis conformation for EPR-free apo 9E5 and in the trans conformation in the EPR-9E5 complex. To the best of our knowledge, this is the first example of a proline cis-trans conformation change occurring in the CDR loop following antigen-antibody binding.

In this study, we performed extensive MD simulations to investigate the mechanism of peptidyl prolyl cis-trans isomerization of the antigen-antibody complex of EPR and 9E5, which was intensively explored for use in cancer therapy^55,56^.

## Results and Discussion

### A stable cis-complex was obtained from 964 independent 200 ns binding MD simulations followed by long MD simulations

**Figure 1:**
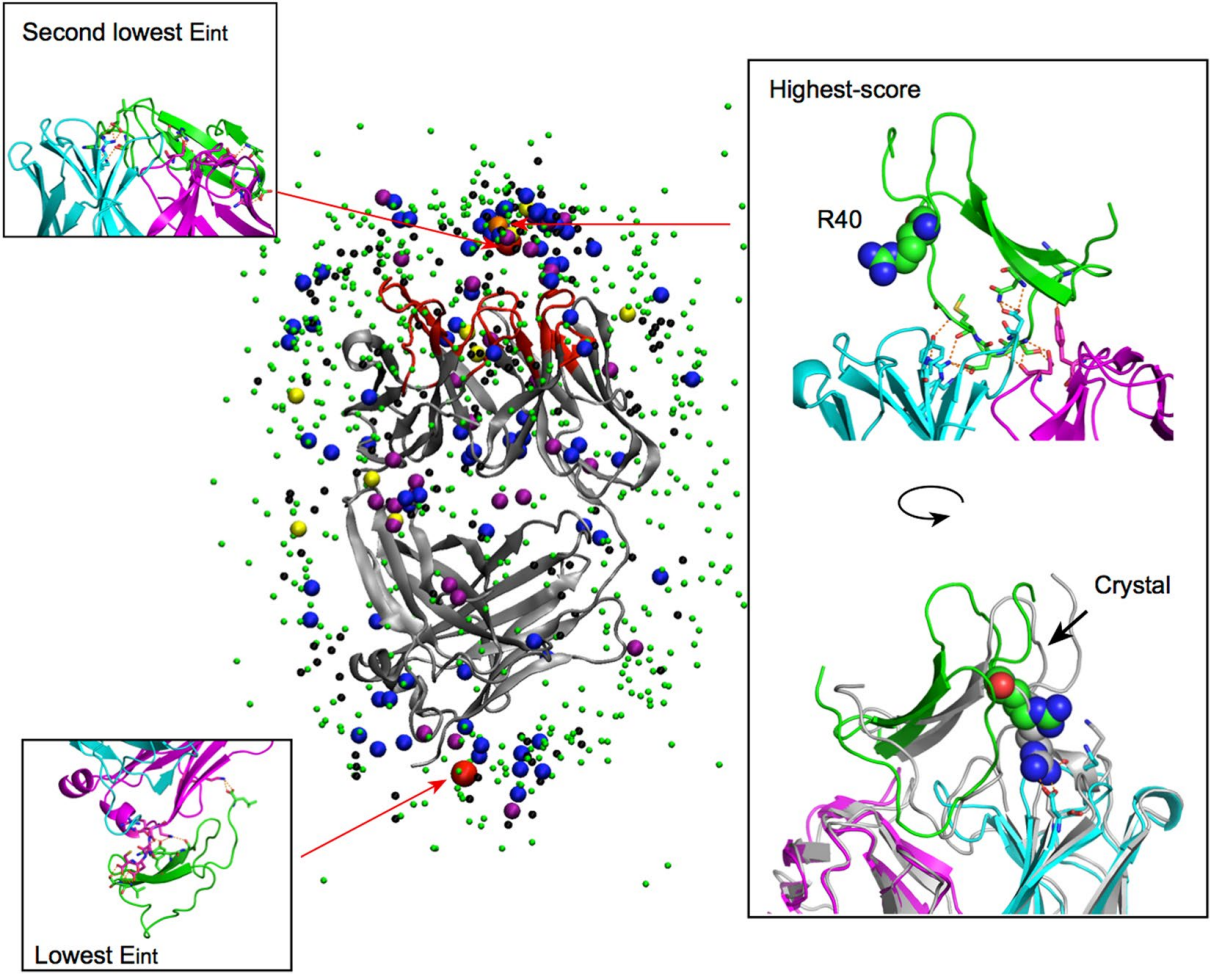
Positions of the EPR COMs on 9E5 observed by binding simulations at 200 ns. The COMs for EPR were classified by the interaction energy (Eint) between EPR and 9E5, and are represented by different colours and circle sizes. Red large circles represent the EPR positions for which Eint was −877 kJ/mol (bottom position) and −810 kJ/mol (approximately the CDR position). Yellow circles, purple circles, blue circles, small black circles, and small green circles represent the positions with the following interaction energies; −800 kJ/mol < Eint ≤ −700 kJ/mol, −700 kJ/mol < Eint ≤ −600 kJ/mol, −600 kJ/mol < Eint ≤ −500 kJ/mol, −500 kJ/mol < Eint ≤ −400 kJ/mol, and −400 kJ/mol < Eint, respectively. The COM of the highest-score structure is represented as an orange circle. 9E5 is represented as a cartoon in grey. CDRs are indicated in red. The EPR-bound structures with the lowest and second lowest Eint, as well as the highest-score structure, are shown as indicated (see inset). The heavy chain, light chain, and EPR in the bound structures are coloured cyan, magenta, and green, respectively. In the figure of the highest-score structure, the crystal structure is also shown in grey (right inset). Interacting residues and hydrogen bonds between EPR and 9E5 are indicated using a stick representation and dashed orange lines, respectively.

To obtain the structure of EPR bound to 9E5 in its apo form and for possible comparison with experimental data by surface plasmon resonance (SPR), we performed 964 independent 200 ns binding MD simulations. We were able to obtain the structures of many complexes between EPR and apo 9E5. Figure 1 shows the EPR centre of mass (COM) on the 9E5 surface at 200 ns. The COMs of the EPR are classified by the interaction energy between EPR and 9E5, which is represented by different colours and circle sizes. At 200 ns, the EPRs were found over a wide region of 9E5, i.e. not only around the CDR region, but also around the constant region of 9E5. This indicates that a time of 200 ns is too short for all EPRs to settle to proper positions. For example, the EPR bound at the constant region of the 9E5 Fab had the lowest interaction energy (averaged over the period of 170 to 200 ns) of −877 kJ/mol. However, the interaction energy of this system was not yet stable, and after additional 2 *μ*s MD simulations, the interaction energy decreased by more than 300 kJ/mol compared with the interaction energy at 200 ns. (Supplementary Fig. S1)

We compared the 964 structures at 200 ns to the x-ray crystal structure, using a similarity score (see Eq. 1 in the Methods section) to select a bound structure. Based on this similarity score, the structure most closely resembling the crystal structure had a score of 0.24. This structure had an interaction energy between the EPR and 9E5 of −584 kJ/mol and was compared to the crystal structure in Fig. 1 (top right). The two structures are similar but have important differences. The main differences are in the CDR-H3 loops and the conformation of Arg40 in EPR. Arg40 in EPR does not form a contact with 9E5 in the highest-score structure, whereas it forms hydrogen bonds with Asp52 and Lys30 in the VH in the crystal structure^55^.

To examine the stability of the highest-score structure in the EPR-apo 9E5 complex, we performed an additional ten independent 2 *μ*s MD simulations using the highest-score structure as the initial configuration. After approximately 1 *μ*s, a bound structure highly similar to the x-ray crystal structure was observed (score = 0.15) in one of the ten simulations. Arg40, which was not found to interact with 9E5 at 200 ns, formed hydrogen bonds with the CDR of 9E5 (specifically at residues Asp52 and Asp31). The interaction energy of this complex between EPR and 9E5 was −1087 kJ/mol (averaged over the period of 1.9 to 2.0 *μ*s), which is lower than that of the highest-score structure by more than 400 kJ/mol. This stable complex between EPR and apo 9E5 is hereafter referred to as the cis-complex.

To examine the stability of the cis-complex, we conducted an additional ten independent 8 *μ*s-MD simulations using the cis-complex structure at 2 *μ*s as the initial configuration. All of the ten trajectories were very stable for 1 *μ*s and then showed small differences (see later subsections). For comparison with the cis-complex, we conducted 3 *μ*s MD simulations starting from the complex crystal structure with trans Pro103, which is referred to as the trans-complex.

### Asp102 is a key residue in 9E5, and EPR also plays an important role in the CDR-H3 loop conformational change from the cis-complex to the trans-complex

**Figure 2:**
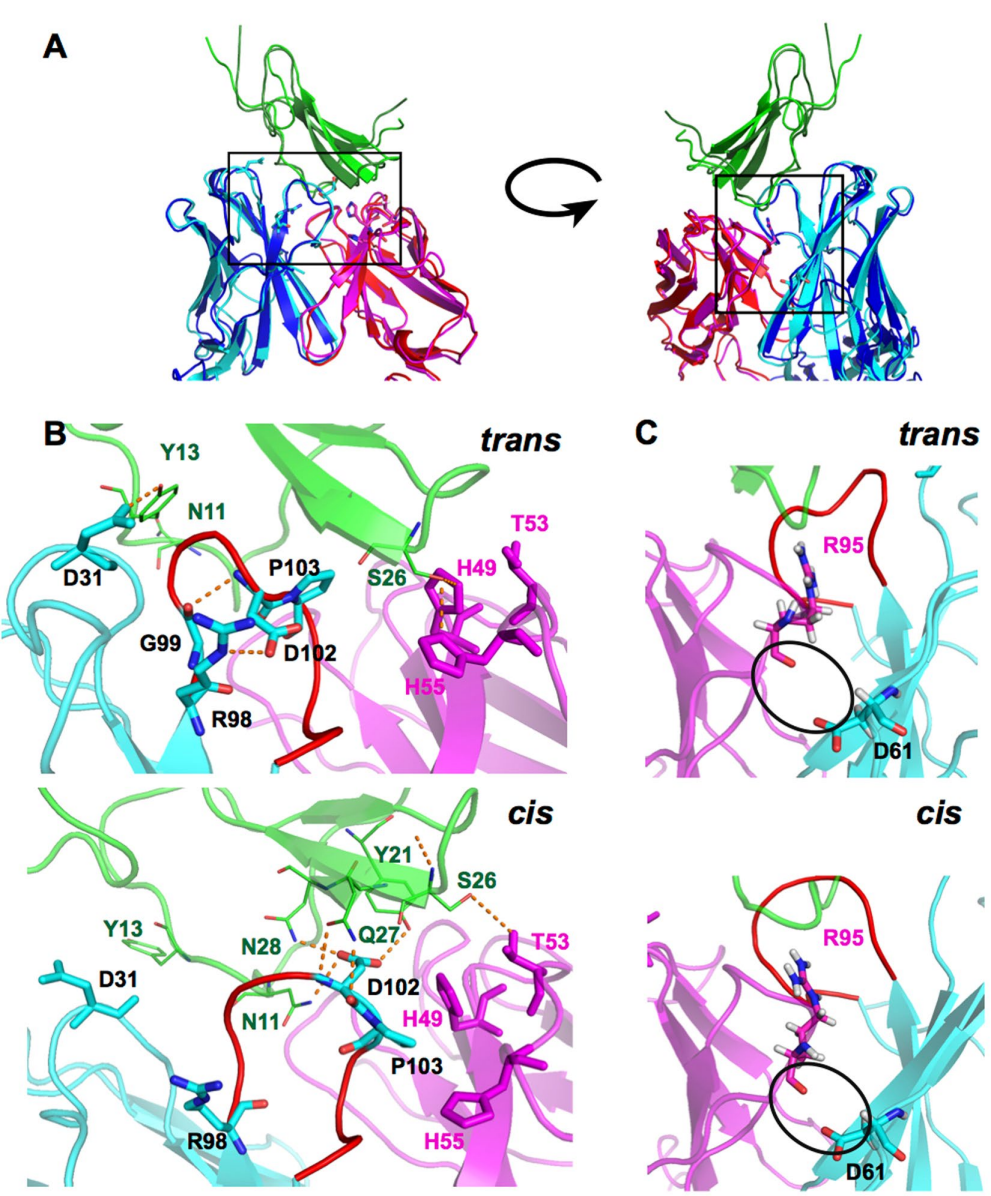
Structural comparison of the CDR-H3 loops and the interface between the heavy and light chains for both the trans-complex and the cis-complex. (**A**) Structures of the trans-complex and the cis-complex. The heavy chain, light chain, and EPR are coloured cyan, magenta, and green for the cis-complex and blue, red, and dark green for the trans-complex, respectively. (**B**) Magnified views of the CDR-H3 loop structures for the trans-complex and cis-complex. The CDR-H3 loop is shown in red. The heavy chain, light chain, and EPR are coloured cyan, magenta, and green, respectively. Hydrogen bonds are shown as orange dashed lines. (**C**) Close-up view of the backside of the CDR-H3 loop and the interface between the heavy chain and the light chain. The distance between Arg95 in the light chain and Asp61 in the heavy chain for the trans-complex is slightly longer than that in the cis-complex, and is represented as black circles. Residues are represented using the one-letter code for amino acids.

Figure 2 shows close-up views of typical CDR-H3 loop structures for the cis-complex and the trans-complex obtained from the 3 *μ*s MD simulations. Conformational differences can be clearly observed in the CDR-H3 loop region; these differences arise as a result of the respective cis and trans conformations of Pro103 in the loop. In addition, the interactions of Asp102, which is adjacent to Pro103, differ between the cis-complex and the trans-complex. In the cis-complex, Asp102 protrudes toward EPR and interacts with several residues in EPR, forming hydrogen bonds with them. In the trans-complex, Asp102 turns toward the base of the CDR-H3 loop and forms hydrogen bonds with Arg98 and Gly99.

We also note a difference at the bottom of the backside of the CDR-H3 loop. As shown in Fig. 2C, in the trans-complex, the distance between Asp61 in the VH and Arg95 in the VL was greater than that in the cis-complex. The minimum distances were 0.49 ± 0.008 nm for the trans-complex and 0.43 ± 0.001 nm for the cis-complex. In addition, Asp61 and Arg95 in the trans-complex do not interact with each other. However, in the case of the cis-complex, there is a very slight repulsive interaction that arises mainly from the oxygen atoms in Asp61 (OD1 and OD2) and the backbone oxygen in Arg95.

Examining the overall structure, we found that the distance between EPR and 9E5 in the cis-complex was slightly larger than that in the trans-complex (the distance between the centre of mass of EPR and that of the Fv fragment of 9E5 was 2.43 ± 0.008 nm for the trans-complex, and 2.47 ± 0.005 nm for the cis-complex). In Fig. 2B, in the cis-complex, Ser26 in EPR interacts with Thr53 in the VL, whereas, in the trans-complex, Ser26 interacts with His49 and His55 in the VL located below Thr53; this was also shown by the x-ray crystallography analysis^55^. These results imply that during isomerisation, VH and VL change their conformations such that the repulsive interaction between them disappears.

**Figure 3:**
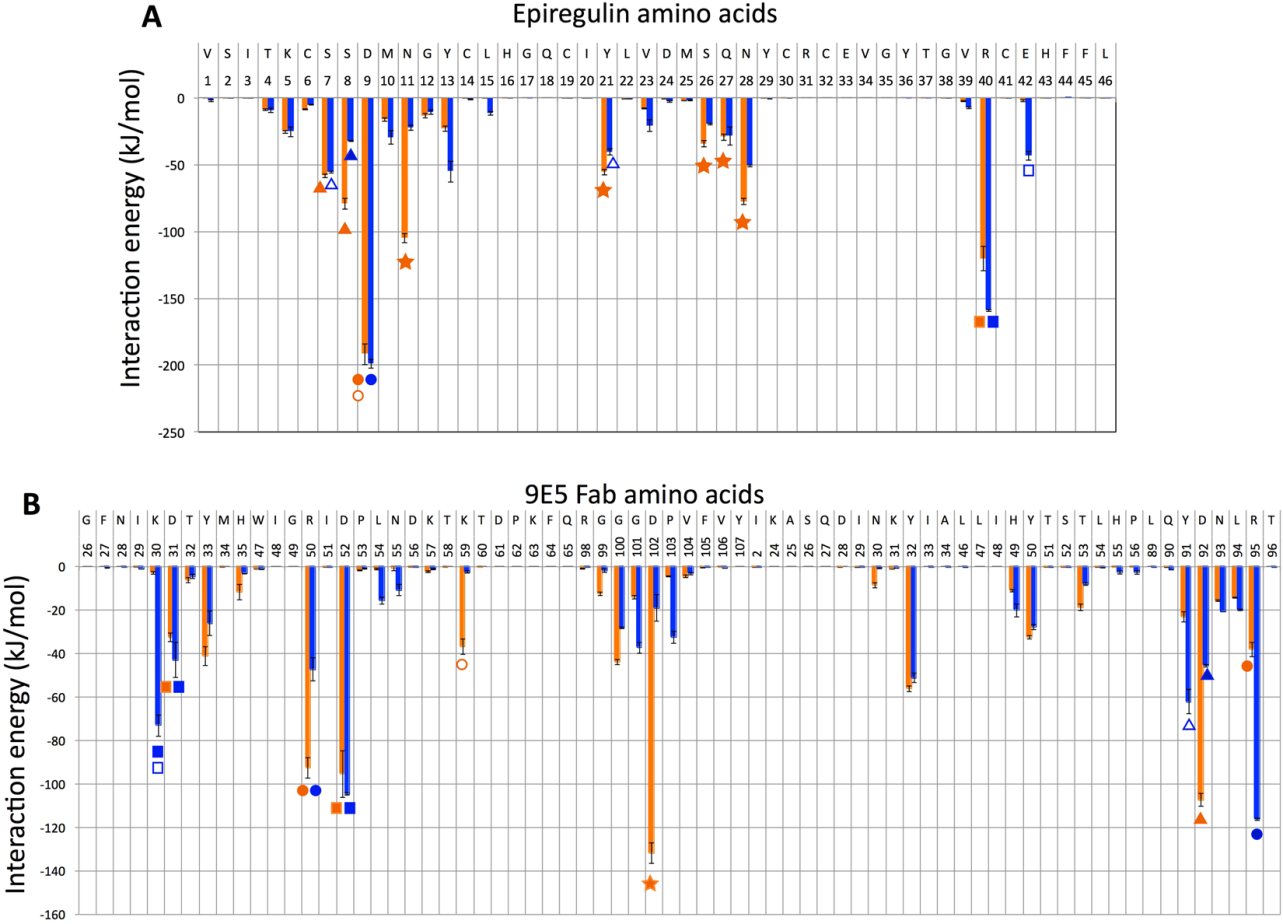
Comparison of a breakdown of the interaction energy between individual residues for the cis-complex and the trans-complex. The calculated interaction energies were averaged over four (trans-complex) or ten (cis-complex) independent trajectories from 2.9 to 3 *μ*s. Orange and blue bars represent the cis-complex and the trans-complex, respectively. Error bars indicate the standard error. The same markers shown at the bottom of the bars indicate residue pairs between 9E5 and EPR interacting with each other. (**A**) Interaction energy between individual EPR residues and the whole 9E5 antibody molecule. (**B**) Interaction energy between individual 9E5 antibody residues and the whole EPR molecule. Residues are represented using the one-letter code for amino acids.

Next, we conducted a detailed analysis of the interaction energies, comparing the cis-complex with the trans-complex, to determine how the interaction energy changes from the transient structure (i.e. the cis-complex) to the final EPR-bound structure (i.e. the trans-complex). Figure 3 shows a breakdown of the interaction energies between each residue in EPR and the entire 9E5 molecule (A) and between each residue in 9E5 and the entire EPR molecule (B). Our previous study revealed three interaction regions in the EPR-9E5 bound structure (trans-complex) obtained from a breakdown of the interaction energies between each residue in EPR and the whole 9E5 molecule. These regions were interaction-1, in which the main interaction is between Asp9 in EPR and Arg50 in VH or Arg95 in VL; interaction-2, in which the main interaction is between Arg40 in EPR and Asp31 and Asp52 in VH; and interaction-3, in which the main interaction is between EPR and the CDR-H3 loop^55^. Among these interactions, interaction-1 was the strongest, and interaction-2 were the next strongest.

These three regions of interaction were also found in the cis-complex as shown in Fig. 3(A), but large differences were noted for all three interactions. First, although the interaction energy between Asp9 in EPR and 9E5 is almost the same in both complexes, the mode of interaction is different. Asp9 interacts with two arginine residues, Arg50 in VH and Arg95 in VL, which are positioned on both sides of Asp9. However, as the direction of the three oxygen atoms (OD1, OD2, and the carbonyl oxygen) in Asp9 is different in both complexes, the main interactions seen for Asp 9 are different. In particular, in the cis-complex, the OD1 and OD2 atoms interact with the guanidinium hydrogen atoms in Arg50, whereas in the trans-complex, the OD1 and OD2 atoms mainly interact with Arg95. Therefore, Asp9 interacts more strongly with Arg50 than with Arg95 in the cis-complex, and vice versa for the trans-complex. In addition, Asp9 in the cis-complex also interacts with Lys59 in the VH. In the trans-complex, Ser7 in EPR interacts mainly with Tyr91 in VL, and Ser8 in EPR interacts mainly with Asp92 in VL, whereas in the cis-complex, both Ser7 and Ser8 interact mainly with Asp92 in VL.

Second, in the trans-complex, Arg40 and Glu42 in EPR have a stronger interaction with the whole 9E5 molecule than that observed in the cis-complex. In the trans-complex but not in the cis-complex, Glu42 in EPR interacts mainly with Lys30 in VH. Third, in the cis-complex, Ser8, Asn11, Tyr21, and Asn28 in EPR strongly interact with the whole 9E5 molecule to a greater extent than in the trans-complex. As shown in Fig. 3(B), three residues (Asn11, Tyr21, and Asn28) among the five residues interact mainly with Asp102 in VH. The interaction energy contributed by Asp102 is very different in both complexes. In the trans-complex, Asp102 interacts strongly with Arg98, which is located in the base of the CDR-H3 loop. However, in the cis-complex, Asp102 interacts with Asn11, Tyr21, Ser26, Gln27, and particularly Asn28 in EPR. This indicates that the dominant mode of interaction for Asp102 changes from an inter-molecular interaction (cis-complex) to an intra-molecular interaction (trans-complex). From these data, it is clear that Asp102 plays a key role in cis-trans isomerisation.

### 10 *μ*s-MD simulations of the cis-complex reveal changes in the omega angle of Pro103

**Figure 4:**
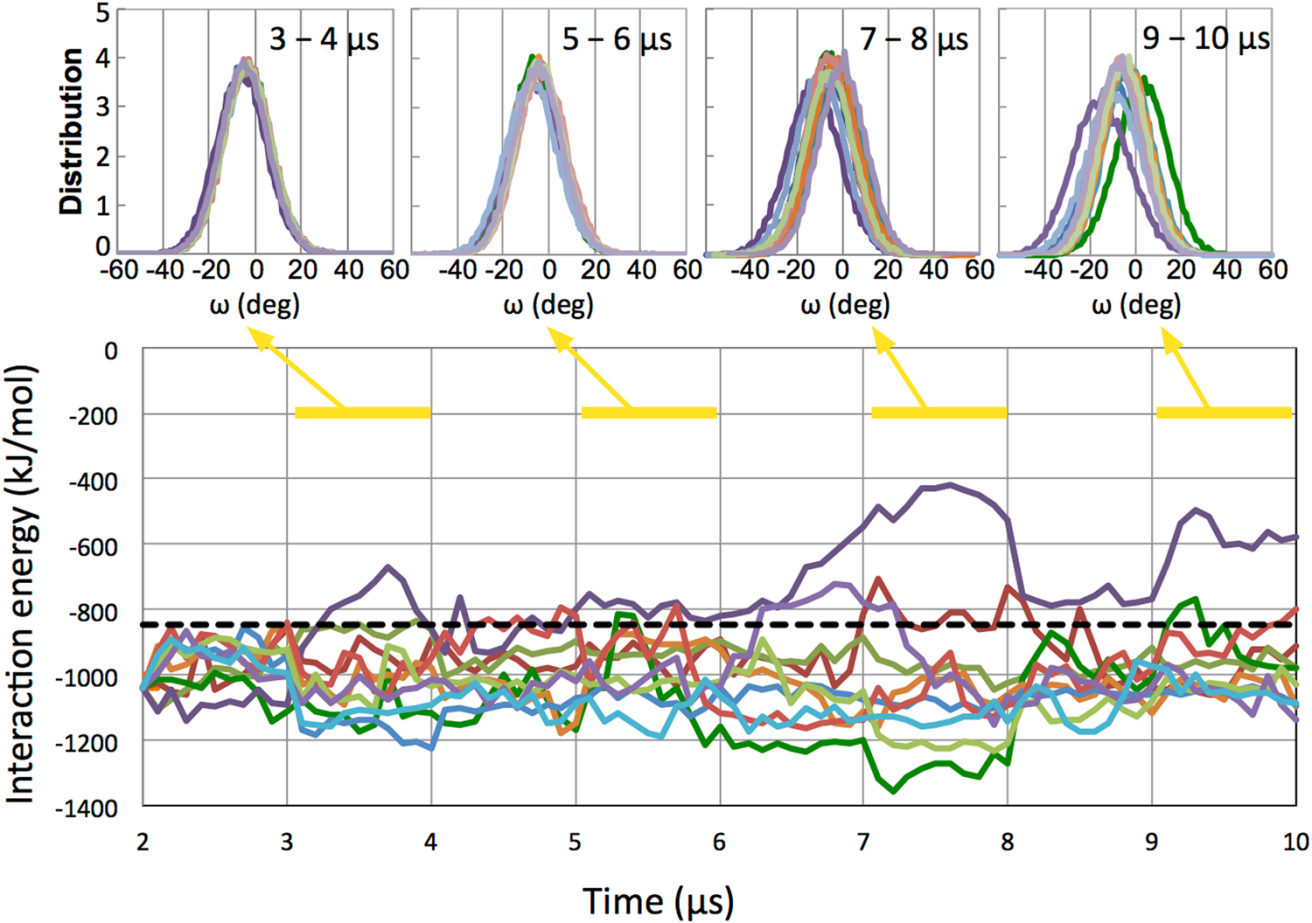
Distribution of *ω* angle and interaction energy between EPR and 9E5 over 10 *μ*s. (Top) Normalized distribution of the omega angle of Pro 103 in the heavy chain over the time intervals of 3–4, 5–6, 7–8, and 9–10 *μ*s. The normalized distribution was computed as follows: the omega angle was measured for each snap shot. Then, the number of each omega angle (bin width of 1 degree) was determined, and finally, this number was divided by the total number of snap shots. (Bottom) Interaction energy between EPR and 9E5. The dashed line indicates the corresponding average value for the four trans-complex trajectories over 2 to 10 *μ*s.

Figure 4 shows changes in the CDR-H3 loop structure and interaction energy of the ten trajectories over time during the 8 *μ*s MD simulations. The cis-complexes over nine trajectories displayed a remarkable conformational stability during the simulations. One trajectory shown in purple revealed that the bound structure underwent a change (score = 0.36), although EPR binding was maintained as described below.

To monitor conformational changes in Pro 103 directly, we calculated the *ω* (omega) dihedral angle of Pro 103, which involves the backbone atoms C*α*-C-N-C*α*. Generally, for the free X-Pro isomer, the *ω* angle will fluctuate around the centre, being close to either 0° or 180°, corresponding to the cis or trans configuration at equilibrium. However, in our system this was not the case. In apo 9E5, the *ω* angle of Pro103 (cis conformer) was found to be distributed around −12° because the C-N bond of Pro103 was twisted in a negative direction due to the strong interaction between Asp102 or Gly101 in VH and His49 in VL (Supplementary Fig. S2). However, in the cis-complex, rather than His49, EPR largely interacted with Asp102 and Gly101 in VH, and the distribution of the *ω* angle was shifted in a positive direction as a result of the interaction; the *ω* angle was found to be distributed around approximately −4°.

The distribution of the *ω* dihedral angle is shown for the same interval time of 1 *μ*s in Fig. 4 (top). For the interval from 3 to 4 *μ*s, and from 5 to 6 *μ*s, the *ω* angles of Pro103 in the ten simulations were distributed in the same way. However, over the time interval of 7 to 8 *μ*s, the distributions in the ten simulations were slightly dispersed. For the time interval from 9 to 10 *μ*s, the distributions of the *ω* angle of two trajectories, shown as purple and green in Fig. 4, markedly shifted in a positive or negative direction, respectively.

The purple trajectory had a change in binding structure after approximately 6.5 *μ*s, such that the hydrogen bond between Arg40 in EPR and Asp31 and Lys30 in 9E5 was disrupted, while at the same time maintaining interaction-1 (Supplementary Fig. S3). In the process, the hydrogen bonds between Arg40 and 9E5 (Asp31 and Lys30) were disrupted, and we observed that the hydrogen bonds broke immediately after Gly101 interacted with His49. The interaction of Gly101 with His49 was observed in apo 9E5. The distribution of the *ω* angle gradually shifted to be more negative at longer times after 6.5 *μ*s, with an average ω angle of −14.8° for the time interval from 9 to 10 *μ*s.

**Figure 5:**
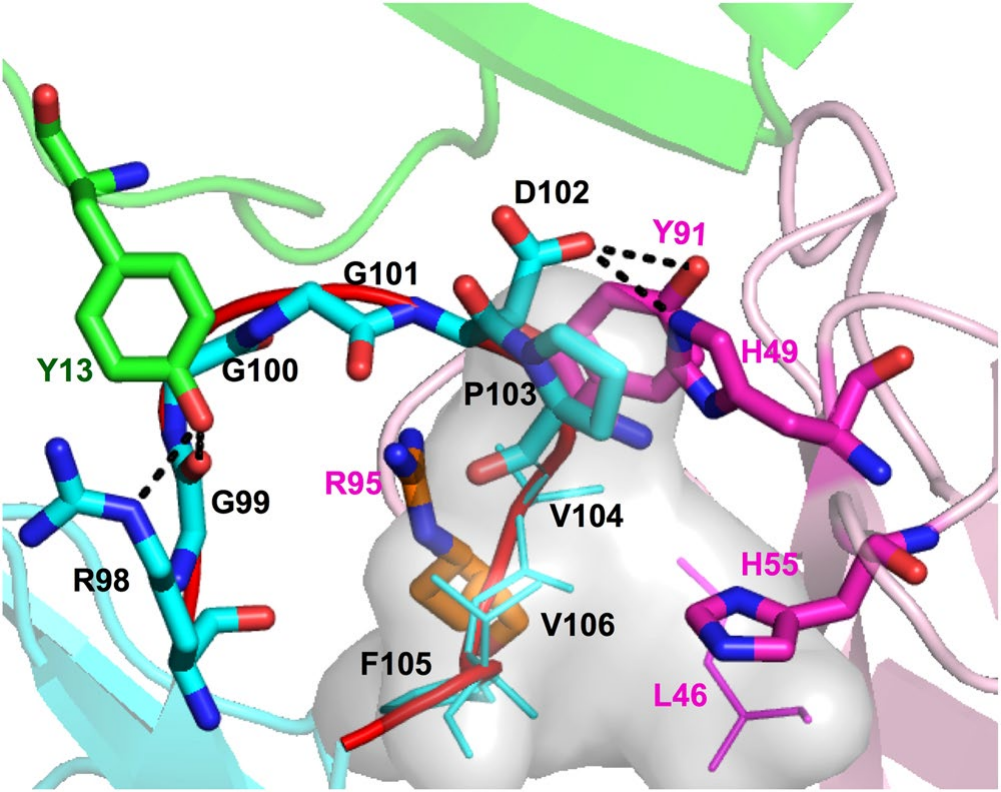
The CDR-H3 loop structure of the cis-complex for the green trajectory at 10 *μ*s. The CDR-H3 loop is shown in red. The heavy chain, light chain, and EPR are coloured cyan, magenta, and green, respectively. The hydrophobic residues Pro103, Val104, Phe105, and Val106 in the CDR-H3 loop and Leu46 in the light chain are shown as a grey surface as well as thin sticks. Leu46 interacts with the hydrophobic residues of the CDR-H3 loop. Hydrogen bonds are shown with black dashed lines. Arg95 (orange stick) in the light chain is predicted to be a residue which may form a contact with Asp102 during isomerisation. Residues are represented using the one-letter code for amino acids.

In contrast, only the green trajectory revealed that the *ω* distribution shifted to the positive side, with the average *ω* angle changing from −6.3° to 2.7°. This small positive *ω* angle implies that the green trajectory did not achieve the transition state for Pro103 required for isomerisation, as the transition state is characterized by a twist of 90 degrees in the *ω* angle of the prolyl peptide bond as has been reported in several previous studies^57,58^. At 10 *μ*s, several major conformational changes in the residues and changes in the interaction mode were observed in the green trajectory. Figure 5 shows a magnified image of the CDR-H3 loop at 10 *μ*s for the green trajectory, and the dashed black lines indicate the newly formed hydrogen bonds between Asp102 and Tyr91 or His49 in VL. The interaction energies between Asp102 and Tyr91 or His49 at 10 *μ*s were larger than those at 3 *μ*s (Table 1). It was also observed that interactions between EPR and Asp102 increased at 10 *μ*s (Table 1). Tyr13 in EPR also had large changes in its mode of interaction. From 9 to 10 *μ*s, Tyr13 in EPR interacts with Gly99 and Arg98 in the CDR-H3 loop through hydrogen bonding, whereas Tyr13 interacts with Tyr33 in VH from 2 to 3 *μ*s.

**Table 1:** Main interaction energies with Asp102 in the green trajectory (kJ/mol). The interaction energies were calculated for 2.9 to 3.0 *μ*s and for 9.9 to 10 *μ*s respectively.

	3 *μ*s	10 *μ*s
**EPR residues**		
Asn11	−19.2	−34.4
Tyr21	−25.5	−56.8
Ser27	−23.8	−26.6
Asn28	−58.7	−66.6
**VL residues**		
His49	−3.2	−28.5
Tyr91	−6.4	−13.2

We performed an additional 10 *μ*s MD simulation for the green trajectory (20 *μ*s in total) to observe the change in the *ω* angle for a longer time.

### Greater interaction of EPR with 9E5 for the cis-complex reflects high association in the SPR experiment

Figure 4 (bottom) shows the time course of the interaction energies between EPR and 9E5 for the ten independent trajectories. During the period from 2 to 10 *μ*s, with the exception of the purple trajectory, the total interaction energies between EPR and 9E5 for the cis-complex were lower than those for the trans-complex (dashed black line); on average, they were more than 100 kJ/mol lower. For the purple trajectory, the total interaction energy decreased mainly due to breakage of the hydrogen bond between Arg40 and 9E5. The average interaction energies from 2 to 10 *μ*s between EPR and 9E5 were −1015 ± 18 kJ/mol for the cis-complex (average of nine trajectories with omission of the purple trajectory) and −846 ± 8 kJ/mol for the trans-complex, respectively (average of four trajectories). The reason why the interaction energy for the cis-complex was so much larger might be found in the different results obtained in the two previous experiments. The binding affinities for EPR with 9E5 (IgG) have been previously measured using two different methods, namely SPR and isothermal titration calorimetry (ITC)^55^. The equilibrium dissociation constant (*K*_*D*_) obtained using SPR (*K*_*D*_ = 0.86 nM) was an order of magnitude stronger than that determined thermodynamically using ITC (*K*_*D*_ = 6.5 nM).

The ITC measurement by Kado *et al*. used a titration interval of approximately 2.5 minutes^55^. Because SPR measures the mass of material binding to the sensor surface, the association rate constant (*K*_*on*_) reflects the initial stage of binding process. The SPR kinetic analysis by Kado *et al*. showed that the calculated *K*_*D*_ is dominated by the high association rate constant, in which the *K*_*D*_ was calculated from *K*_*D*_ = *k*_*off*_/*k*_*on*_. Therefore, it can be concluded that the large calculated interaction energy for the cis-complex gave the high association rate constant between ERP and 9E5.

### The environment around the CDR-H3 loop is suitable for isomerisation

Previous computational studies of the cis-trans isomerisation in the PPIase system have shown that the substrate-binding sites of a protein (enzyme) in which isomerisation of the substrate occurs have two features, i.e. several polar residues leading to the C-N bond rotation of proline in the substrate, and a hydrophobic pocket for substrate association^2^. These electrostatic and hydrophobic features seen in the substrate-PPIase system can also be seen in the CDR-H3 loop of 9E5. In this case, Gly99-Gly100-Gly101-Asp102-Pro103 in the CDR-H3 loop correspond to the substrate for the PPIase. With respect to the polar residues around the CDR-H3 loop, there are several EPR residues that interact strongly with Asp 102 in the cis-complex, as described above. A hydrophobic region, consisting of Val104, Phe105, Val106 in VH and Leu46 in VL, is located around the CDR-H3 loop and is represented as a grey surface in Fig. 5.

Two other factors also promote isomerisation in the present system: (1) Pro103 and Asp102 are confined in the loop structure, and (2) three consecutive glycines are present in the CDR-H3 loop. With respect to Pro103 and Asp102 in the loop structure, a relationship between loop form and an increase in the isomerisation rate was previously reported, in which the rate of cis-trans isomerisation of the *ω* angle was enhanced by a factor of 10 by constraining the prolyl peptide bond in a loop conformation^59,60^. These studies suggest that conformational constraints by the loop form lead to increased conformational flexibility of the peptide backbone and lead to the conformation of the prolyl peptide in the transition state. Additionally, the three consecutive glycines present in the CDR-H3 loop make this region flexible. It has also been previously shown that the beta-hairpin loop, in which the Gly-Gly motif is constrained, provides increased flexibility of Gly-Gly and rapid isomerisation^48^. Interestingly, in our study, the fluctuation of Gly99 in the CDR-H3 loop was greater in the cis-complex than in apo 9E5 (Supplementary Fig. S4). Constraint of the CDR-H3 loop by EPR increases the flexibility of Gly99, and this increased flexibility would be convenient to increase the rate of Pro103 cis-trans isomerisation. From these data, it can be concluded that surrounding Pro103 in the CDR-H3 loop there is an environment that enables cis-trans isomerisation to occur much more rapidly compared to that in free X-Pro peptides.

### Catalysis mechanism for cis-trans isomerisation in the EPR-9E5 system

Cis-trans isomerisation occurs through rotation of the C-N bond. There are two directions by which the C-N bond can be rotated: clockwise and anti-clockwise looking from the N-terminal to the C-terminal direction along the C-N bond. It should be noted that a positive omega angle corresponds to moving the N-terminal atom anti-clockwise or the C-terminal atom clockwise. However, in this case, the rotation of the C-N bond that occurs during isomerisation is in a predominately unidirectional fashion, i.e., in the direction that moves the N-terminal C *α* atom of Asp102 anti-clockwise, because the residues that interact with Asp102 to help promote the isomerisation are not exposed to the solvent side, but rather are exposed to the inner side of 9E5. Therefore, the side chain of Asp102 can rotate more easily anti-clockwise (the omega angle being positive) than clockwise. As mentioned above, we observed a green trajectory that had a positive omega angle for Pro103 over the interval of 9 to 10 *μ*s. The positive value was observed from approximately 8 to 14 *μ*s during 20 *μ*s (Supplementary Fig. S5).

**Figure 6:**
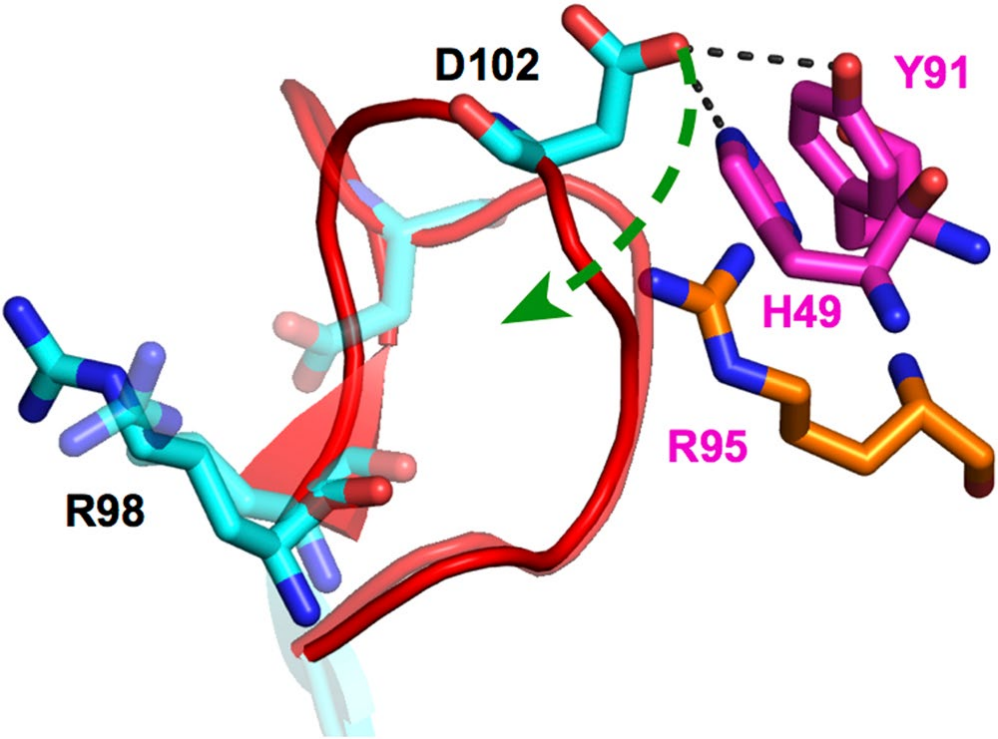
Predicted pathway for cis-trans isomerisation in the CDR-H3 loop. Arg95 in the VL is coloured in orange. Asp102 interacting with Tyr91 and His49 in the VL then interacts with Arg95, and rotates to bind to Arg98 in the VH. The structure of the trans-complex is also shown as transparent sticks and a cartoon. Residues are represented using the one-letter code for amino acids.

Regarding Asp102, the charged residue Arg95 in VL is close to Tyr91, which is interacting with Asp102 (Fig. 5). From these data, we envision the cis-trans isomerisation process in the EPR-9E5 antibody system occurring as follows (Fig. 6): Asp102 interacting with Tyr91 and His49 at 10 *μ*s next interacts with Arg95 in VL. Subsequently, the C-N bond of Pro103 rotates to allow binding of Asp102 to Arg98 in the CDR-H3 loop. This interaction involves guanidino groups in Arg98 forming hydrogen bonds with oxygen in Asp102, thus completing the isomerisation process. The time course of the minimum distance between the Asp102 and Arg95 in the green trajectory change was found to be correlated with the *ω* angle during 20 *μ*s (Supplementary Fig. S5). The minimum distance was reduced from 0.7 at 2 *μ*s to 0.5 nm at 10 *μ*s as the *ω* angle of Pro103 shifted to the positive side. Arg95 may also facilitate rotation of the Pro103 peptide bond by stabilizing the transition state of the isomerisation. The role of Arg95 is therefore similar to that of the highly conserved Arg55 found in CypA^42,61^, in which Arg55 in the substrate-binding site of CypA promotes isomerisation by weakening the double-bond character of the C-N bond by forming hydrogen bonds between the guanidino group in Arg55 and the carbonyl oxygen of the proline substrate.

From our data, we conclude that the EPR-9E5 structure seen in the green trajectory at 10 *μ*s is in a stable intermediate state in over a microsecond time scale, leading to the transition state for isomerisation. This result implies that several stable intermediate states are adopted until isomerisation is complete, and the intermediate states are determined by the interactions between Asp102 and its surrounding residues. Thus, in this case isomerisation occurs by rotating Asp102 while changing the interacting partner residue in 9E5. At the same time, with respect to the increased interaction between EPR and Asp102 seen at 10 *μ*s, EPR helps to stabilize the intermediate states during the isomerisation process. Thus, we consider that the VL and EPR cooperate to catalyse the cis-trans isomerisation of Pro103 like PPIase.

### Binding free energy and total free energy including conformation changes

The cis-complex has lower binding free energy than the trans-complex because the interaction energies between EPR and 9E5 are favourable for the cis-complex, as explained in the previous subsections, and the fluctuation of the CDR-H3 loop was larger in the cis-complex (as shown in Supplementary Fig. S4). The larger fluctuation increases the entropy, which is favourable for the binding free energy. The difference in binding free energy between the cis-complex and trans-complex was measured as the difference of the dissociation constants (*K*_*D*_) between SPR and ITC.

The cis-complex is a transient state and the trans-complex is the final stable binding structure of EPR and 9E5. This means that the total free energy of the system should be favourable to the trans-complex. We expected some structural stress in the cis-complex. As mentioned in the previous section, there was a slight repulsive interaction between the residues in the VH-VL domain interface as shown in Fig. 2C. To clarify the detailed structural differences among the cis-complex, trans-complex and apo 9E5, we examined Ramachandran angles (*ϕ* and *ψ*) of all residues of 9E5 and analysed the angle distributions between 2 *μ*s and 3 *μ*s in the MD simulations. Excluding some residues near the binding region around the CDR, we found that several residues had different Ramachandran angle distributions. A typical such residue was Asn160 in heavy chain constant region 1 (CH1). This residue is located far from the binding region, but its conformation in the cis-complex differed from that in the trans-complex or apo 9E5, although the conformation of Asn160 was almost same between apo 9E5 and the trans-complex (Supplementary Fig. S6). It is not clear whether the conformational difference of Asn160 was related to an unfavourable total free energy of the cis-complex, but the conformation difference disappeared with the trans isomerisation of Pro103.

The interaction energy of the green trajectory changed near 15 *μ*s in the MD simulation to be smaller than that of the trans-complex (Supplementary Fig. S7). This implies that the binding free energy would also be smaller. Finally, the cis-complex would change to a trans-complex having a lower total free energy. Considering that Pro103 is in a loop structure of the CDR-H3 including flexible consecutive glycines, we expected that the cis-trans isomerisation would occur on the order of hundreds of microseconds.

## Methods

### MD simulation

**Figure 7:**
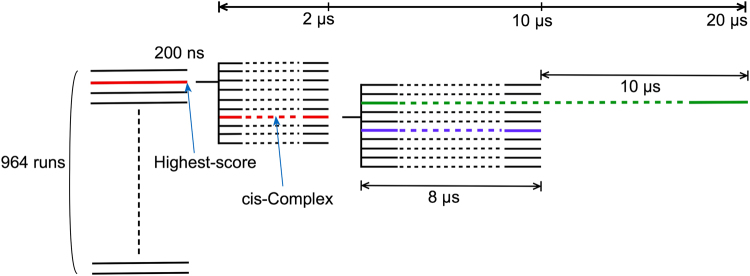
A schematic figure for the MD simulations which started from the free EPR and apo form of 9E5.

Figure 7 schematically shows the MD simulations that started from the free EPR and apo form 9E5 explained in the Results and Discussion section. For comparison, we conducted four 10 *μ*s MD simulations for the EPR-9E5 complex (i.e. the trans-complex) and apo 9E5, in which the starting structures for the apo 9E5 and the trans-complex were taken from the x-ray crystal structures^55^. We also performed an additional 10 *μ*s MD simulation for the green trajectory (20 *μ*s in total) mentioned in the previous subsection.

Simulations of EPR binding to apo 9E5 (binding MD simulations) were performed by placing EPR at a minimum distance of 0.9 nm from the CDR of apo 9E5. *Na* ^+^ and *Cl* ^−^ ions were then added to make a neutral solution of 0.15 M. After energy minimization, the heavy atoms in the protein were restrained for 200 ps using a harmonic potential with a force constant of 1000 *kJ* ^−1^ *nm* ^−2^ to relax the water molecules. Subsequently, MD simulations were performed under isotropic isothermal-isobaric (NPT) conditions. MD simulations were conducted with different initial velocities assigned to each atom of the simulation system from a Boltzmann distribution. A Nosé-Hoover thermostat^62,63^ was used to keep the temperature at 298 K, with a relaxation time of 1 ps. A Parrinello-Rahman barostat^64,65^ was used to isotropically regulate the pressure at 1 atm, with a relaxation time of 1 ps. The simulation time step was 4 fs and a virtual site model was adopted to remove the bond-angle degrees of freedom from hydrogen atoms. The leap-frog algorithm for integrating the equations of motion and the particle mesh and the Ewald (PME) method^66,67^ for calculating electrostatic interactions with a real-space cutoff of 1.0 nm were used. The neighbour list cut-off was set to 1.0 nm. The Lennard-Jones potential was smoothly switched to zero over the range of 0.8–0.9 nm. All bond lengths for proteins were constrained using the LINCS algorithm^68^. The atomic coordinates were saved every 5000 steps (20 ps) for analysis. All-atom MD simulations described in this study were performed using GROMACS version 4.6.5 (for the 964 MD binding simulations) and version 4.6.7 (for long-term simulations)^69^ with Fuji force field^70^ for proteins, AMBER models for ions and TIP3P for water^71^.

### Similarity analysis

To select a bound structure similar to the x-ray crystal structure from the many complexes obtained at 200 ns, we analysed the similarity to the x-ray crystal structure, using a similarity score defined by

1 $$score=\sqrt{\frac{\sum _{pair\mathrm{=1}}^{{N}_{pairs}}{({r}_{pair}-{r}_{pair}^{crystal})}^{2}}{{N}_{pairs}}}$$

where *r*_*pair*_ represents the minimum distance between an EPR residue and a 9E5 residue of a pair for the bound structure from binding simulations, and ${r}_{pair}^{crystal}$ represents that of the crystal structure. The residue pairs used for the score calculations were taken from Table 2 of Kado *et al*.^55^. The total number of selected residue pairs (*N*_*pair*_) was 19.

### Data Availability

The datasets generated and analysed during the current study are available from the corresponding author on reasonable request.

## Electronic supplementary material

Supplementary information
