# Supplementary material for: Initiation of prolyl cis-trans isomerisation in the CDR-H3 loop of an antibody in response to antigen binding
Source: Sci Rep. 2017 Dec 5;7:16964. doi: 10.1038/s41598-017-16766-8 (PMC5717248; doi:10.1038/s41598-017-16766-8)
Supplement: Supplementary file 1 — Supplementary information [file 41598_2017_16766_MOESM1_ESM.doc]

**Supplementary Information**

Initiation of prolyl cis-trans isomerisation in the CDR-H3 loop of an antibody in response to antigen binding

Keiko Shinoda1 and Hideaki Fujitani*

1Laboratory of Systems Biology and Medicine, Research Center for Advanced Science and Technology, The University of Tokyo, 4-6-1 Komaba, Meguro-ku, Tokyo, 153-8904, Japan

(a) (b)


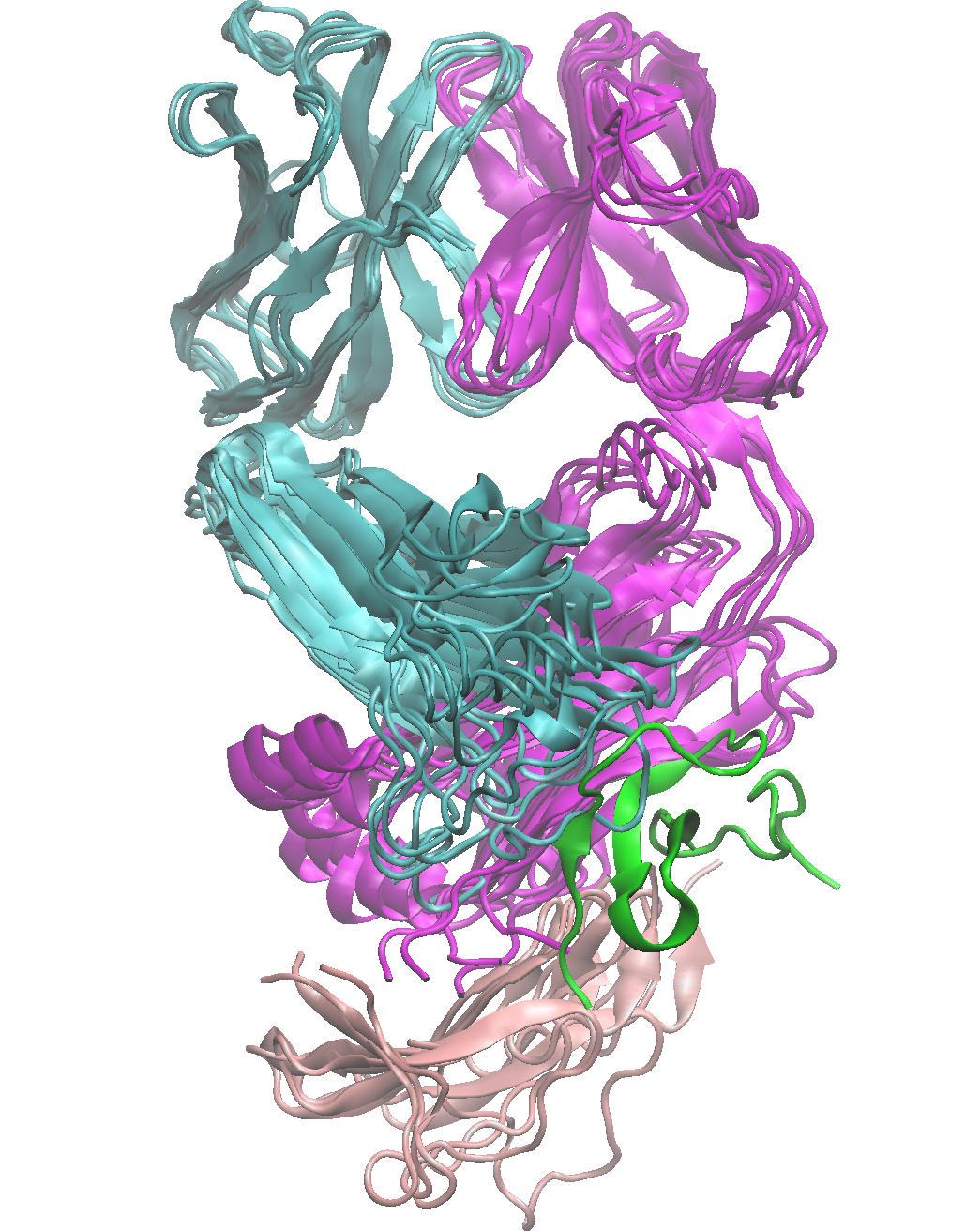

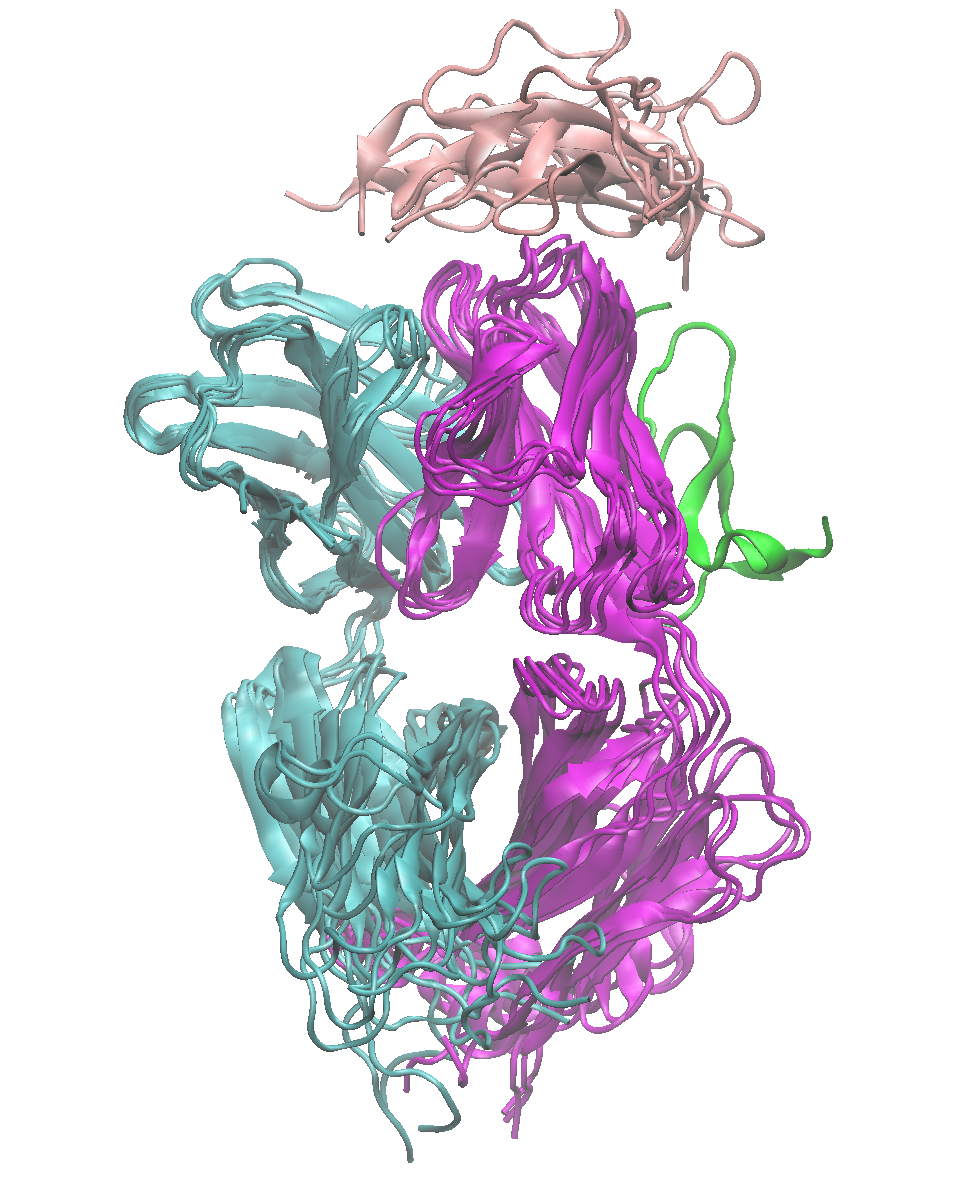


**Figure S1. After 2μs configurations: 2μs-MD simulations of the lowest (a) and second lowest (b) E_int_ systems**

The heavy chains and light chains are colored cyan and magenta, respectively. The EPRs are colored pink or green.

To evaluate how stable the lowest and second lowest (-810 kJ/mol) E_int_ systems are, we have conducted additional 2 μs five independent MD simulations for each system. For the both systems, four of fifth simulations reveal the almost same EPR-bound structures during 2 μs, whereas for one simulation (EPRs are colored green), the EPR move 2.6 and 3.1 nm from the other averaged COM for the lowest and second lowest interaction energy systems, respectively. The interaction energies averaged over 1 to 2μs are small more than 300 kJ/mol, of -542 ± 63 and -499 ± 23 kJ/mol, compared with those at 200 ns.

1. (b)

**Figure S2.** **The normalized distribution of the omega angle of Pro 103 in the VH for apo 9E5 system (a) and the CDR-H3 loop structure of apo 9E5 (b).** Plotted for distributions correspond to four apo 9E5 trajectories, which have different initial velocity. The normalized distribution was computed as same manner in Figure 4. Residues are represented using the one-letter code for amino acids.

**Figure S3. Interaction energy between EPR residues and 9E5 for “purple” trajectory**

Orange bars represent the interaction energies averaged from 2.9 to 3.0 μs, and cyan bars is that for 9.9 to 10μs, respectively. Residues are represented using the one-letter code for amino acids.

**Figure S4. The root mean square fluctuations (RMSFs) of backbone atoms of the region Ala92-Thu112 including CDR-H3 loop.**

The orange, blue, and green lines represent the RMSF for the cis-Complex, trans-Complex, and apo, respectively. The RMSFs were calculated for 2 to 3 μs after the fitting of backbone atoms of the base region of the loop (Ala92-Ser97, Gly111-Val116) with respect to that of a snapshot at 2 μs. Error bars indicate the standard errors of average for five (apo and trans-Complex) or ten (cis-Complex) trajectories for 2 to 3 μs. Residues are represented using the one-letter code for amino acids.

**Figure S5. Omega angle of Pro103 and Distance between Asp102 of VH and Arg95 of VL**

The blue and black lines represent the omega angle and minimum distance between Asp102 and Arg95, respectively.


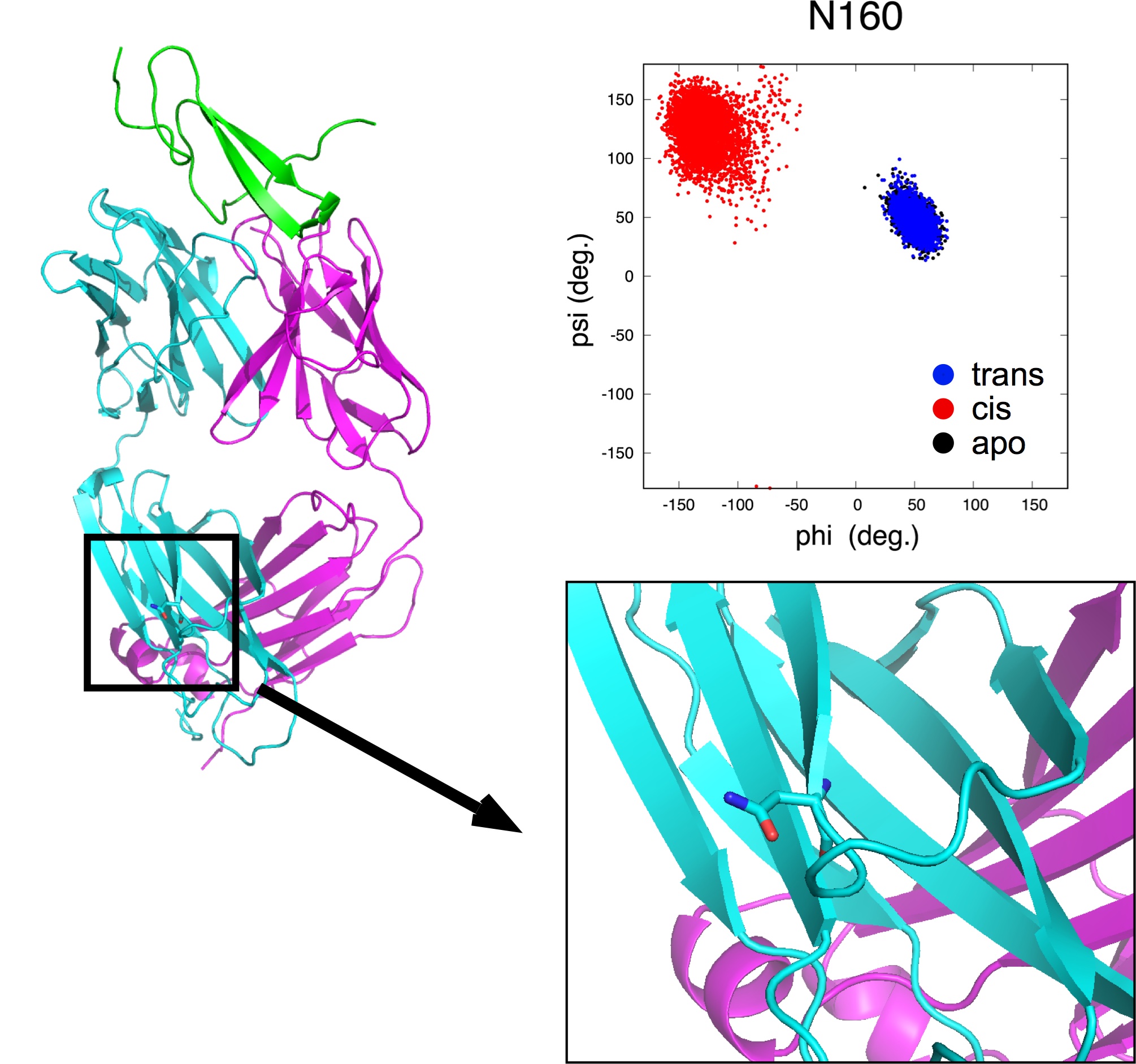


**Figure S6. Phi and psi dihedral angles of Asn160 which is located in the constant region of 9E5**

The blue, red and black dots represent phi (x-axis) and psi (y-axis) dihedral angles of Asn160 in the trans-complex, cis-complex, and apo structure, respectively. Phi and psi angles of Asn160 were calculated every 100 frames in four trajectories for 2-3 μs in each system.


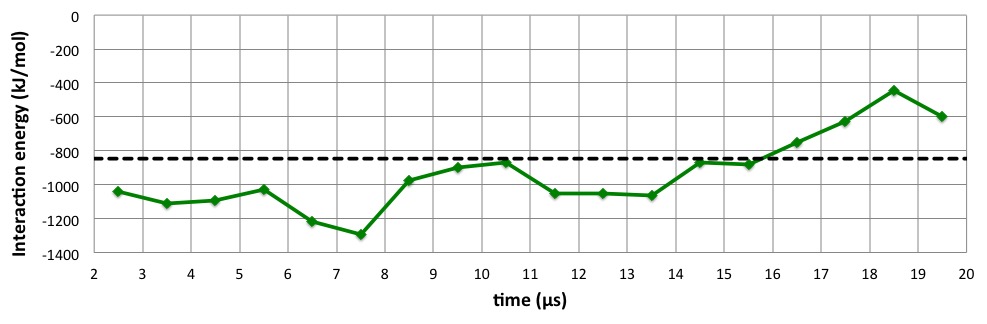


**Figure S7. Interaction energy between EPR and 9E5 for green trajectory**

The green line indicates the interaction energy between EPR and 9E5 for the green trajectory and the black dashed line indicates the average interaction energy for the four trans-complex trajectories over 2 to 10 μs.
